# TP53 protein levels, RNA-based pathway assessment, and race among invasive breast cancer cases

**DOI:** 10.1038/s41523-018-0067-5

**Published:** 2018-06-25

**Authors:** Lindsay A. Williams, Ebonee N. Butler, Xuezheng Sun, Emma H. Allott, Stephanie M. Cohen, Ashley M. Fuller, Katherine A. Hoadley, Charles M. Perou, Joseph Geradts, Andrew F. Olshan, Melissa A. Troester

**Affiliations:** 1 Department of Epidemiology, Gillings School of Global Public Health, University of North Carolina at Chapel Hill, Chapel Hill, NC 27599 USA; 2 Department of Nutrition, Gillings School of Global Public Health, University of North Carolina at Chapel Hill, Chapel Hill, NC 27599 USA; 3 Lineberger Comprehensive Cancer Center, University of North Carolina at Chapel Hill, Chapel Hill, NC 27599 USA; 4 Department of Pathology and Laboratory Medicine, School of Medicine, University of North Carolina at Chapel Hill, Chapel Hill, NC 27599 USA; 5 Department of Genetics, University of North Carolina at Chapel Hill, Chapel Hill, NC 27599 USA; 6 Department of Pathology, Dana-Farber Cancer Institute, Boston, MA 02115 USA

## Abstract

Mutations in tumor suppressor *TP53* have been inconsistently linked to breast cancer risk factors and survival. Immunohistochemistry (IHC) staining, a primary clinical means of *TP53* mutation determination, only detects mutations that facilitate protein accumulation (e.g., missense mutations). RNA-based pathway methods capture functional status and may aid in understanding the role of TP53 function in racial disparities of breast cancer. *TP53* status was assessed among invasive breast cancer cases from the Carolina Breast Cancer Study (CBCS) (2008–2013) using IHC and an established RNA-based TP53 signature (CBCS and The Cancer Genome Atlas (TCGA)). Frequency of TP53 status (IHC, RNA-based) was estimated in association with tumor characteristics, PAM50 intrinsic subtype, age, and race using relative frequency differences (RFDs) and 95% confidence intervals (95% CI) as the measure of association. Approximately 60% of basal-like tumors were TP53 protein positive (IHC), while nearly 100% were TP53 mutant-like (RNA). Luminal A tumors had low frequency of TP53 positivity (IHC: 7.9%) and mutant-like status (RNA: 1.7%). Mutant-like TP53 (RNA) was strongly associated with age ≤50 years, high tumor grade, advanced stage of disease, large tumor size, and basal-like and HER2 intrinsic subtypes. Black race was strongly associated with TP53 mutant-like status (RNA*)* (RFD: 24.8%, 95% CI: 20.5, 29.0) even after adjusting for age, grade, stage (RFD: 11.3%; 95% CI: 7.6, 15.0). Associations were attenuated and non-significant when measured by IHC. IHC-based TP53 status is an insensitive measurement of TP53 functional status. RNA-based methods suggest a role for TP53 in tumor prognostic features and racial disparities.

## Diagnostics: RNA test linked to prognostic features and racial differences

RNA-based assays offer a more sensitive and clinically informative measure of mutations in the tumor suppressor *TP53* among women with invasive breast cancer than do immunohistochemistry techniques that can only detect altered proteins. Using tumor samples from more than 1000 women enrolled in the Carolina Breast Cancer Study (CBCS), Melissa Troester from the University of North Carolina at Chapel Hill, USA, and coworkers assessed the functional status of *TP53* via both classical immunohistochemistry methods and an RNA-based test of expression levels among 52 *TP53*-dependent genes. The results of the RNA analysis were strongly associated with younger age-of-onset, higher grade tumors, more advanced stage disease, larger tumor size, aggressive cancer subtypes and race—with more black women harboring *TP53* mutant-like tumors than white women. By comparison, these associations were weaker or non-significant when using immunohistochemistry-based tests.

## Introduction

The tumor suppressor gene TP53 is mutated in 30–40% of breast tumors, with variation in mutation frequency by intrinsic subtype and race.^1–14^ Up to 80% of basal-like and 70% of human epidermal growth factor 2-enriched (HER2-enriched) breast tumors harbor *TP53* mutations, which commonly include nonsense and frame shift alterations.^1^ Mutations occur at much lower frequencies among Luminal A (12–23%) and Luminal B (15–29%) tumors^1,2,9,13–15^ and are primarily missense mutations in form. In addition, *TP53* mutation rates are higher among black women (25–32%) compared to white women (7–23%),^11,16^ which may potentially help to explain existing racial disparities in breast cancer incidence and survival. Many previous studies that have evaluated race and TP53 status have used immunohistochemistry (IHC) methods that detect missense mutations resulting in protein accumulation. Moreover, recent analyses based on The Cancer Genome Atlas (TCGA) data also detected racial differences in the frequency of *TP53* mutations based on DNA sequence.^1,17^

Recently, several studies have used RNA-based methods to determine TP53 functional status. This approach avoids some functional misclassification that could result from the low sensitivity of IHC and the low specificity of TP53 sequence mutations for detecting functional defects in the *TP53* pathway.^18–20^ Using this approach, we applied a validated, 52-gene signature^18^ to evaluate the RNA expression of *TP53*-dependent genes, classifying 1013 invasive breast tumors from Phase 3 of the population-based CBCS as TP53 mutant-like or wild-type-like. RNA-based TP53 status was compared to IHC status (*n* = 1291 total, *n* = 843 RNA and IHC), and both were evaluated in association with race, age, tumor characteristics, and PAM50 intrinsic subtype. Results were placed in context of parallel analyses of TCGA data using the same TP53-dependent gene expression signature and whole genome DNA sequencing data.

## Results

The frequency of TP53 protein overexpression (IHC) and TP53 mutant-like status (RNA) within categories of age, race, and selected tumor characteristics is presented in Table 1. Significant differences in TP53 protein overexpression were observed for grade, stage, tumor size, and PAM50 subtype. ER−, PR−, HER2+, and Triple Negative (ER−/PR−/HER2−) tumors more frequently overexpressed TP53. There were no differences in TP53 protein overexpression by age or lymph node status.

**Table 1:** Associations between patient and tumor characteristics and TP53 status (IHC, RNA) in CBCS3

	IHC average weighted percent Positivity ≥10%				NanoString TP53 gene expression signature			
	Mutant (≥10%)	Wild type (<10%)			Mutant	Wild type		
	*N* (%^a^)	*N* (%^a^)	RFD^b^ (95% CI)	*p*-value	*N* (%^a^)	*N* (%^a^)	RFD^b^ (95% CI)	*p*-value
Total	312 (21.3)	979 (78.7)			491 (40.9)	522 (59.1)		
Age								
≤50 years	145 (29.8)	461 (28.0)	1.8 (−1.7, 5.3)	0.31	263 (34.4)	247 (28.6)	5.8 (2.4, 9.2)	<0.01
>50 years	167 (70.2)	518 (72.0)	Ref.		228 (65.6)	275 (71.4)	Ref.	
Grade								
Low-Intermediate	65 (28.6)	620 (75.0)	Ref.		102 (26.2)	392 (82.2)	Ref.	
High	232 (71.4)	295 (25.0)	46.4 (42.8, 49.9)	<0.01	381 (73.8)	108 (17.8)	56.0 (52.9, 59.1)	<0.01
Missing	15	64			8	22		
Stage								
I, II	259 (84.3)	848 (88.9)	Ref.		392 (80.3)	449 (87.2)	Ref.	
III, IV	51 (15.7)	118 (11.1)	4.5 (1.8, 7.2)	<0.01	98 (19.7)	68 (12.8)	6.9 (4.2, 9.6)	<0.01
Missing	2	13			1	5		
Node Status								
positive	108 (33.8)	363 (32.7)	1.1 (-2.5, 4.7)	0.54	222 (44.3)	204 (36.6)	7.7 (4.1, 11.2)	<0.01
negative	202 (66.2)	614 (67.3)	Ref.		267 (55.7)	317 (63.4)	Ref.	
missing	2	2			2	1		
Tumor Size								
≤2 cm	159 (57.1)	570 (64.9)	Ref.		199 (44.4)	306 (64.3)	Ref.	
>2 cm	149 (42.9)	382 (35.1)	7.8 (0.4, 11.5)	<0.01	288 (55.6)	208 (35.7)	19.9 (16.3, 23.5)	<0.01
missing	4	27			4	8		
ER Status								
positive	118 (46.6)	800 (86.7)	Ref.	<0.01	207 (44.6)	487 (96.5)	Ref.	
negative	190 (53.4)	153 (13.3)	40.1 (36.5, 43.7)		280 (55.4)	27 (3.5)	51.9 (49.0, 54.8)	<0.01
missing	4	26			4	8		
PR Status								
positive	83 (33.9)	634 (69.1)	Ref.	<0.01	140 (29.8)	408 (82.5)	Ref.	
negative	223 (66.1)	316 (30.9)	35.2 (31.6, 38.9)		343 (70.2)	103 (17.5)	52.7 (49.5, 55.8)	<0.01
missing	6	29			8	11		
HER2 Status								
negative	251 (83.9)	821 (88.4)	Ref.		379 (79.8)	469 (94.0)	Ref.	
positive	54 (16.1)	117 (11.6)	4.5 (1.8, 7.3)	<0.01	101 (20.2)	38 (6.0)	14.2 (11.7, 16.7)	<0.01
missing	7	41			11	15		
HR+/Her2−								
ER+ or PR+/HER2−	90 (45.7)	707 (89.1)	Ref.		152 (43.2)	448 (97.1)	Ref.	
ER-/PR-/HER2−	158 (54.3)	112 (10.9)	43.3 (39.4, 47.2)	<0.01	224 (56.8)	21 (2.9)	53.8 (50.6, 57.1)	<0.01
missing	64	160			115	53		
PAM50 Subtype								
Luminal A/B	59 (30.1)	441 (74.5)	Ref.		111 (22.5)	476 (91.8)	Ref.	
Basal-like, HER2, Normal-like	161 (69.9)	182 (25.5)	44.4 (40.2, 48.7)	<0.01	380 (77.5)	46 (8.2)	69.3 (66.6, 71.9)	<0.01
missing	92	356						

^a^Percentages weighted for sampling design, models adjusted for sampling design

^b^RFD: Relative frequency difference

The patterns of association observed for TP53 protein overexpression were qualitatively similar, but stronger in magnitude when using RNA-based methods (Table 1). TP53 mutant-like status was associated with age ≤50 years, high tumor grade, higher stage of disease, node positive disease, larger tumors (>2 cm), ER−, PR−, HER2+, Triple Negative (ER-/PR-/HER2-) tumors, and non-Luminal PAM50 subtypes (basal-like, HER2-enriched, Normal-like). Comparing RNA-based calls to IHC-based calls, 77.7% of IHC mutant/TP53 over-expressing tumors were mutant-like by RNA; however, many tumors that appear to be TP53 wild type by IHC have RNA-based profiles suggestive of TP53 loss (223 of 623; 30%) (Supplemental Table 2).

Table 2 presents TP53 status (IHC and RNA-based), overall and stratified by PAM50 subtype (CBCS3 and TCGA). In CBCS3, more tumors had TP53 mutant-like status (41.0%) than TP53 overexpression (21.3%). The increased frequency of mutant-like status by RNA-based methods was observed among basal-like (RNA: 99.4% vs. IHC: 58.1%), Luminal B (RNA: 44.3% vs. IHC: 16.3%), and HER2-enriched (RNA: 95.8% vs. IHC: 29.4%) subtypes. Lower frequencies of TP53 mutant-like status using RNA were observed among Luminal A (RNA: 1.7% vs. IHC: 7.9%), and normal-like tumors (RNA: 19.9% vs. IHC: 21.5%). Compared to DNA-based methods, RNA-based methods appeared to detect a higher proportion of TP53 mutant-like tumors in TCGA (52.6% mutant-like compared to 35.2% mutant by DNA sequencing). In subtype-stratified analyses, RNA-based assessments detected more mutant-like tumors relative to DNA sequencing among basal-like (RNA: 98.2% vs. DNA: 89.6%), Luminal A (RNA: 25.4% vs. DNA: 11.7%), Luminal B (RNA: 78.4% vs. DNA: 33.1%), and HER2-enriched (RNA: 86.8% vs. DNA: 68.0%) tumors, but not among normal-like tumors (RNA: 40.5% vs. DNA: 45.2%).

**Table 2:** Frequency of TP53 mutation by PAM50 subtype and technical method in CBCS3 and TCGA

		Overall	Basal-like	Luminal A	Luminal B	HER2-enriched	Normal-like
Study	Method	*N* (%^a^) TP53 mutant	*N* (%^a^) TP53 mutant	*N* (%^a^) TP53 mutant	*N* (%^a^) TP53 mutant	*N* (%^a^) TP53 mutant	*N* (%^a^) TP53 mutant
CBCS3	IHC	312 (21.3)	120 (58.1)	29 (7.9)	30 (16.3)	34 (29.4)	7 (21.5)
	RNA-based	491 (41.0)	256 (99.4)	11 (1.7)	100 (44.3)	114 (95.8)	10 (19.9)
TCGA	RNA-based	475 (52.6)	167 (98.2)	123 (25.4)	124 (78.4)	46 (86.8)	15 (40.5)
	DNA-based missense mutations	164 (20.2)	65 (42.2)	32 (7.5)	33 (22.3)	22 (44.0)	12 (38.7)
	any mutation	285 (35.2)	138 (89.6)	50 (11.7)	49 (33.1)	34 (68.0)	14 (45.2)
	wild-type	526 (64.9)	16 (10.4)	378 (88.3)	99 (66.9)	16 (32.0)	17 (54.8)

^a^Percentages weighted for sampling design

**Fig. 1:**
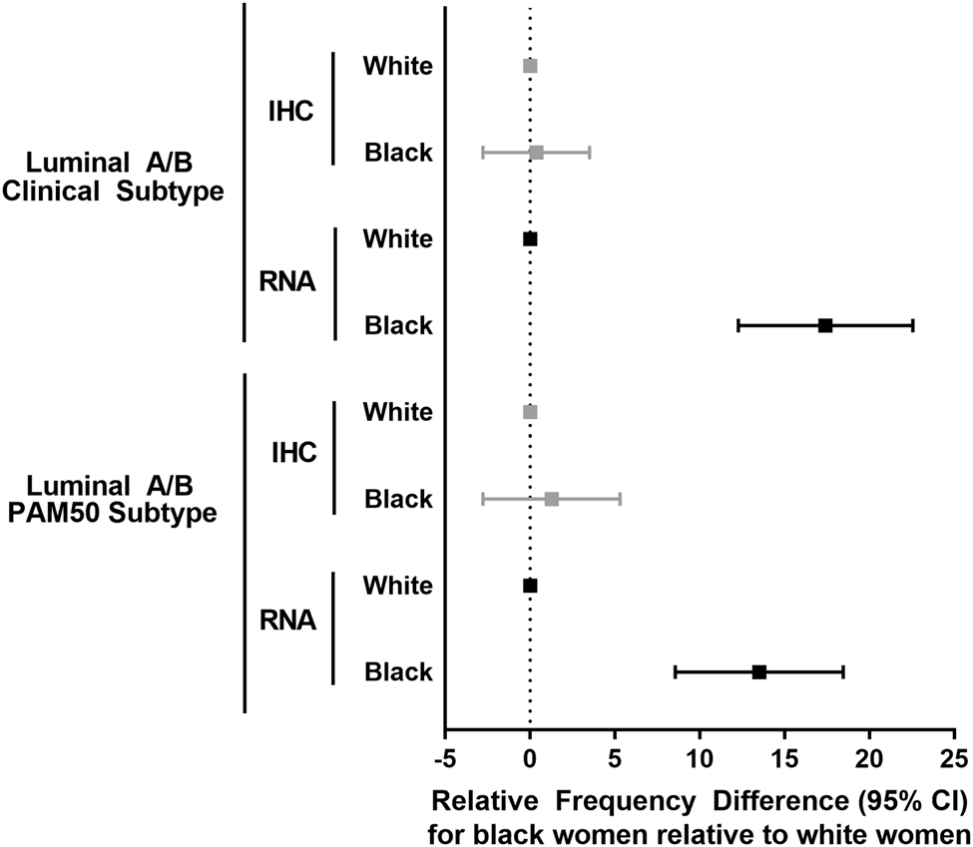
Relative frequency differences (RFDs) and 95% confidence interval (95% CI) for TP53 mutation status as determined by IHC and RNA among black women compared to white women in Luminal A/B clinical and PAM50 subtype cases only

Black race was strongly associated with TP53 status in CBCS3 and TCGA (Table 3), particularly for the RNA-based signature. Among Luminal A/B tumors, by both clinical subtype or PAM50 subtype, black women were significantly more likely to have TP53 mutant-like status when measured by RNA (black vs. white RFD: 17.4%; 95% CI: 12.3, 22.6 for clinical subtype; black vs. white RFD: 13.5%; 95% CI: 8.5, 18.5 for PAM50 subtype) (Fig. 1). These differences were not evident when considering TP53 status measured by IHC. In univariate analyses of CBCS3 data, black women had a significantly higher proportion of TP53 overexpression and TP53 mutant-like status than white women (IHC: RFD: 11.0%, 95% CI: 7.6, 14.5; RNA: RFD: 24.8%, 95% CI: 20.5, 29.0). TCGA RNA-based results also showed higher frequency of TP53 mutant-like status in black women (RFD: 19.9%, 95% CI: 12.0, 27.9). Upon adjustment for age and stage in TCGA, and age, stage, and grade in CBCS3, TP53 mutant-like status remained significantly associated with black race. However, the association between TP53 mutant status and black race in both TCGA and CBCS3 was not statistically significant by IHC (CBCS3) or RNA (CBCS3, TCGA) when accounting for PAM50 intrinsic subtype along with the other covariates.

**Table 3:** Association between race and TP53 expression and mutation status by technical method, CBCS3 and TCGA

	White	Black	Univariate^b^		Multivariate I		Multivariate II	
Method	(*N*,%)	(*N*,%)	RFD (95% CI)	*p*-value	RFD (95% CI)	*p*-value	RFD (95% CI)	*p*-value
CBCS3^a^								
IHC								
wild type	490 (81.2)^a^	425 (70.2)^a^	Ref.		Ref.		Ref.	
mutant	117 (18.8)^a^	180 (29.8)^a^	11.0 (7.6, 14.5)	<0.01	2.8 (−0.2, 5.8)^c^	0.07	−0.3 (−3.4, 2.9)^e^	0.86
RNA-based								
wild type	316 (64.1)^a^	184 (39.4)^a^	Ref.		Ref.		Ref.	
mutant	183 (35.7)^a^	300 (60.6)^a^	24.8 (20.5, 29.0)	<0.01	11.3 (7.6, 15.0)^c^	<0.01	3.2 (−1.6, 7.9)^e^	0.19
TCGA								
RNA-based								
wild type	372 (51.2)	51 (31.3)	Ref.		Ref.		Ref.	
mutant	354 (48.8)	112 (68.7)	19.9 (12.0, 27.9)	<0.01	19.3 (11.4, 27.3)^d^	<0.01	2.3 (−6.6, 11.2)^f^	0.61

^a^Percentages weighted for sampling design, CBCS3 models adjusted for sampling weights

^b^Univariate analyses restricted to same set of participants in multivariate model I

^c^Adjusted for age, stage and grade

^d^Adjusted for age and stage

^e^Adjusted for age, stage, grade, and PAM50 subtype

^f^Adjusted for age, stage, and PAM50 subtype

To explore whether TP53-race associations were robust to differences in age, we further evaluated associations between tumor subtype and TP53 status after cross-classifying on both age and race and three-marker clinical subtypes (Supplemental Table 3). Among IHC-based Triple Negative (ER−, PR−, HER2−) tumors, black women ≤50 years of age had a higher frequency of TP53 protein overexpression (63.7%) and TP53 mutant-like status (95.0%) than white women ≤50 years of age (IHC: 46.5%; RNA: 87.2%) and black and white women >50 years of age (black women >50 IHC: 60.0%; RNA: 88.8%; white women >50 IHC: 56.8%; RNA: 94.8%). After restricting these analyses to the more clinically homogenous group of ER+ or PR+, or hormone receptor-positive (HR+), and HER2- tumors, race and age remained statistically significantly associated with RNA-based TP53 mutant-like status with black women ≤50 years of age having the highest frequency of mutant-like status (39.9%), but this difference did not persist for TP53 protein overexpression. HER2-positive tumors were unable to be evaluated in these additional analyses due to low sample size.

## Discussion

In our study, nearly twice as many breast tumors were classified as TP53 mutant-like when using RNA-based gene expression compared to IHC, consistent with the observation that IHC methods miss many mutations that are not associated with TP53 protein overexpression. This improved sensitivity to detect functional defects in the TP53 pathway resulted in stronger associations with almost all clinical and demographic variables, including age, grade, stage, node status, tumor size and molecular subtype. When using TCGA data to compare RNA-based classification of TP53 status to DNA-based mutation detection, the gene expression signature is also more sensitive, in line with the observation that some defects in the TP53 pathway arise from other genetic events not captured by TP53 protein expression alone (e.g., MDM2 amplification). All of the changes in TP53 gene dosimetry (amplification and deletion) and all relevant regulators of *TP53* expression and function, such as *MDM2, p63, p21* (both up and downstream), are incompletely understood, so we cannot confirm that all tumors showing RNA-based mutant-like status have underlying genetic or epigenetic defects in the pathway. However, the TP53 signature used for this analysis has been validated in TCGA analyses,^1^ and is based on human cell line studies with isogenic knockdown of TP53.^18^ Moreover, patterns of association between molecular subtype and TP53 status observed in the CBCS were similar to patterns observed in TCGA, with almost all basal-like tumors and very few Luminal A tumors showing mutant-like TP53 signatures or DNA-based mutations.

One metric for assessing the utility of the RNA-based signature is to assess whether the signature recapitulates some of the patterns of TP53 mutation by clinical variables. Indeed, our results are consistent with several previous studies. We and others have found that higher grade tumors are more frequently classified as TP53 mutant by DNA sequencing and protein expression.^14,21–23^ Additionally, we observed RNA-based mutant-like TP53 status was more common among cases diagnosed with node positive or higher stage of disease, as reported by others using DNA or IHC-based methods.^14,23–25^ Tumors greater than 2 cm in size also more frequently overexpressed TP53 and were more frequently TP53 mutant-like, similar to what has been reported previously.^14,23^ However, our results also show that the relative sensitivity of the TP53 signature may afford some advantages. For example, RNA-based TP53 mutant-like status was associated with younger age (≤50 years), but TP53 overexpression was not. In previous studies, age has been inconsistently linked to TP53 status.^14,21^ It is possible that other inconsistencies in previous studies may in part lie in misclassification of TP53 functional status.

Given the established mortality disparities by race and the prognostic value of *TP53* mutation status,^15,26^ our primary hypothesis was that TP53 functional defects would be more common with black race. We found that black women had higher frequencies of *TP53* mutations and TP53 mutant-like status than white women, consistent with previous studies.^1,11,12,16,17,25,27^ Further, we observed a significant difference in the relative frequencies of TP53 mutant-like status for black compared to white women when restricted to Luminal A/B clinical and PAM50 subtypes. Differences were imprecise and not significant when comparing black and white women for Triple Negative or basal-like tumors as these subtypes had over 90% of black and white women classified as TP53 mutant by both IHC and RNA (results not shown). Given a large sample size of black women (*n* > 500 in CBCS3 compared to ~150 in TCGA), we were able to evaluate age and race simultaneously in CBCS3, showing that young, black women have the highest rates of TP53 mutant-like status when compared to young white women and older white and black women.

Our study should be viewed in light of some limitations. First, the IHC measurements were taken from multiple TMA cores and not whole slide images. While participants could have up to four cores sampled from a single tissue block, there could be some differences in the percent positivity for the whole tissue versus the averaged percent positivity that we calculated weighted by core cellularity. All sample top and bottom tissue block slides were analyzed by study pathologists to ensure that the sample was of invasive disease, but we cannot exclude the possibility of normal epithelium or DCIS being counted by the automated algorithm for TP53 positivity. While possible, we anticipate little impact on the final results from this phenomenon. Regarding IHC, we employed a positive stain approach where binding of the TP53 antibody would signal a mutation in TP53 resulting in accumulation of the protein within the nucleus. There are other staining methods that could help to identify the mutations that truncate theTP53 protein, but those were not applied here. Similarly, to determine RNA gene expression for the tissue samples two cores were taken and pooled for the PAM50 analysis. The use of multiple cores from different locations in the tumor may or may not be representative of the gene expression of the tumor as a whole. We believe that using multiple cores made our gene expression profiles of the tumor more comprehensive, but this precludes us from studying heterogeneity in gene expression within the tumor. Finally, we did not have sufficient sample size with RNA data, nor sufficient recurrence events to justify analyses of TP53-dependent survivorship. Future work is planned to complete RNA-based assessment of TP53 for the remainder of the CBCS3 cohort. In addition, we are continuing to follow patients for recurrence and survival so that the role of TP53 in outcome disparities, overall and according to intrinsic subtype, can be assessed.

The methods and findings presented here show that the RNA-based method we applied to detect functional defects in TP53 is more sensitive than IHC and yields similar associations with subtype and race as previously observed in other datasets, but with greater sensitivity and stronger associations relative to IHC methods. Future work should evaluate etiologic and survival associations for TP53 based on RNA-based signatures particularly by race where black women with ER+, HER2- disease have worse outcomes than white women.^28,29^ An advantage of the current method is that measuring RNA through methods like NanoString assays can simultaneously measure tumor subtype (e.g., PAM50) and TP53 mutant-like status in both fresh frozen and FFPE samples. Given recent findings that the prognostic value of TP53 may depend upon subtype,^14^ future analyses integrating subtype and TP53 status will help ascertain the role of this pathway in breast cancer outcomes and race-ethnicity mortality disparities.

## Methods

### Study population

The present analysis includes invasive breast cancer cases from the population-based CBCS, Phase 3 (CBCS3) (2008-2013). Methods for the CBCS have been described in detail elsewhere.^2,28,30–33^ Briefly, eligible cases were women aged 20–74 years with a first diagnosis of invasive breast cancer, identified via rapid case ascertainment through the NC Central Cancer Registry. The current analysis is restricted to cases who had tumor tissue available for IHC analysis (*N* = 448), RNA isolation from formalin fixed paraffin embedded (FFPE) tissue (*N* = 170), or both (*N* = 843). A total of 1461 of the 2998 cases in CBCS3 were included. All study procedures were approved by the University of North Carolina (UNC) School of Medicine Institutional Review Board and participants provided written informed consent.

TCGA population has been described previously.^1,7^ A total of 903 participants included in this analysis were enrolled from various medical centers, provided informed consent for access to tumor tissue, and had RNA expression data available. All TCGA samples were processed under the approval of the respective Institutional Review Boards and participants provided written informed consent.

### TP53 status by IHC

Immunohistochemistry staining conditions were optimized using breast tissue sections and human cell lines with established *TP53* mutation status [(wild type: MCF-7, SUM102), (p.R175H mismatch mutant: SKBR3)]. All cell lines were purchased from the American Type Culture Collection and low-passage cultures were prepared, with mycoplasma testing performed regularly and confirmed negative. IHC was carried out at the UNC Translational Pathology Laboratory using a Bond Autostainer (Leica Microsystems Inc. Norwell, MA 02061). Slides were dewaxed in Bond Dewax solution (AR9222) and hydrated in Bond Wash solution (AR9590). Antigen retrieval was performed for 20 min in Bond-Epitope Retrieval Solution 1 pH-6.0 (AR9961). Slides were incubated for 15 min with mouse monoclonal anti-TP53 antibody (BioGenex, Fremont, CA; clone D07 [catalog # MU239-UC], 1:7200). Detection was performed using the Bond Intense R Detection System (DS9263) supplemented with Dako EnVision Mouse (Carpinteria, CA, K4001). Stained slides were counterstained with hematoxylin, dehydrated, and coverslipped. A control tissue microarray (TMA) containing TP53 positive and negative breast tissue and cell lines was included in each run along with a negative control (no primary antibody). CBCS3 TMA construction has been previously described.^34^ TMAs were constructed with 1–4, 1.0-mm cores per participant.

TP53-stained TMA slides were scanned using the Aperio ScanScope XT at 20× magnification. Details of the scoring algorithm have been described previously.^35^ Briefly, TP53 staining was measured with the Aperio Nuclearv9 algorithm by quantifying the tumor cellularity and was combined with the Genie Histology Pattern Recognition tool to correctly classify the number of tumor and normal epithelial cells per core allowing for enrichment of tumor cells. Algorithm parameters including nuclear size and nuclear compactness were optimized to achieve the best nuclear segmentation. The algorithm returned a total number of nuclei per core and the number of nuclei positive for TP53. To determine the average percent positivity, a method of core-to-case collapsing developed by Allott et al.^34^ was used by summing the total number of nuclei/core (1–4 cores/participant). Each core was given a weight equal to the number of core nuclei divided by the total nuclei for the participant. For the core weighted percent positivity, the core’s TP53 percent positivity was multiplied by the core’s weight. The weighted core values were summed to obtain the participant’s overall weighted percent TP53 positivity. Weighted percent TP53 positivity was dichotomized to classify patients as negative or positive (<10% for negative/wild-type, ≥10% positive/mutant). The ≥10% cut point was selected based on the best sensitivity, specificity, and accuracy measures.

### Clinical characteristics of tumors

Data for estrogen receptor (ER), progesterone receptor (PR), and human epidermal growth factor receptor 2 (HER2) status, nodal status, and stage of disease were abstracted from medical records for CBCS3 participants. For CBCS3, tumor grade was defined by central pathologist review by the CBCS study pathologist.

### PAM50 intrinsic subtype and TP53 status [RNA (CBCS3, TCGA) and DNA-based (TCGA)]

In CBCS3, NanoString assays were conducted on 1122 samples from 1042 cases as described previously.^34,36^ Briefly, hematoxylin and eosin stained slides were reviewed by a pathologist and 1–9 1.0-mm cores were sampled from tumor regions of corresponding blocks. Cases selected for NanoString were randomized to three batches and technicians were blinded to tumor characteristics and clinical data. Intrabatch QC data were reported previously^36^ and showed high correlation among batch controls. RNA was isolated using the Qiagen RNeasy FFPE kit (cat# 73504). NanoString gene expression experiments, which use RNA counting as a measure of gene expression, were conducted in the UNC Translational Genomics Laboratory.

We excluded 39 samples with insufficient quality as identified by the NanoStringNorm package in Bioconductor and 18 samples from participants with two different tumor blocks. There were 52 patients with multiple samples from the same tumor block. Gene expression data for these participants was calculated as the RNA average of the samples if the correlation coefficients for expression were >0.90; otherwise the sample with better quality score from NanoStringNorm was used. A total of 1013 cases were included in the final analysis.

Two signatures were evaluated to classify patients: (1) the previously validated 52-gene *TP53*-dependent signature^18^ and (2) a 50-gene PAM50 signature.^36^ For the TP53 signature, mutant-like vs. normal-like class was determined based on a similarity-to-centroid approach.^18^ For PAM50 subtype, samples were categorized into one of five intrinsic subtypes (Luminal A, Luminal B, basal-like, HER2-enriched, and Normal-like) as described in Parker et al. ^36,37^

For TCGA, *TP53* DNA mutations were determined (missense mutation, any mutation) using the mutation annotation file (MAF) from the TCGA breast lobular manuscript page (https://tcga-data.nci.nih.gov/docs/publications/brca_2015/). RNA sequencing expression data from flash frozen invasive tumor tissue samples was used to classify patients according to the PAM50 and TP53 signatures.^7,37^

### Statistical analysis

Participant and tumor characteristics for the CBCS3 study population overall, as well as the subsets with IHC (*n* = 1, 291) and NanoString data (*n* = 1013) can be found in Supplemental Table 1. The IHC subset included all samples with TMAs available, while the NanoString subset represented a random sampling of CBC3 participants with at least two tumor tissue cores remaining after TMA construction. The IHC subset differed significantly from CBCS3 as a whole by age at diagnosis, stage of disease, and tumor size. The NanoString dataset differed from CBCS3 overall by grade. The IHC and NanoString subset differed significantly from one another by lymph node status, tumor size, and tumor grade.

While the RNA-based data from CBCS3 was compared to similar data from TCGA, patients in TCGA were more frequently older, more frequently white, had larger tumors, and had high grade tumors (results not shown).

Generalized linear models were used to estimate relative frequency differences (RFDs) and corresponding 95% Confidence Intervals (95% CI) as the measure of association between TP53 status and variables of interest.^38^ Sample size counts are unweighted and percentages are weighted to account for the sampling design of CBCS3, which oversampled black and younger women. Models are also adjusted for the sampling weights to account for the study design. The following variables were studied in association with TP53 status: age at diagnosis ( ≤ 50, > 50), race (self-report black, non-black ( > 98% white, referred to as white)), tumor grade (low-intermediate, high), stage of disease (I/II, III/IV), lymph node status (positive, negative), tumor size ( ≤ 2 cm, > 2 cm), PAM50 intrinsic breast cancer subtype (Luminal A, Luminal B, HER2-enriched, basal-like, Normal-like),^37^ ER status (negative, positive), PR status (negative, positive), and HER2 status (negative, positive). For subtype analyses, Luminal A/B was selected as the reference group. RFDs by race are presented as univariate and multivariate-adjusted (CBCS3: age, stage, grade, and PAM50 subtype; TCGA: age, stage, and PAM50 subtype). All analyses were done in SAS version 9.4 (SAS Institute, Cary, NC). *P*-values were two-sided with an alpha value for statistical significance equal to 0.05.

### Data availability

TCGA data can be found here: https://tcga-data.nci.nih.gov/docs/publications/brca_2015/. The CBCS3 datasets generated and/or analyzed during the current study are not publicly available due to some human subjects restrictions, but may be available from the corresponding author on reasonable request.

## Electronic supplementary material

Supplemental Tables 1-3
