# Supplementary material for: TP53 protein levels, RNA-based pathway assessment, and race among invasive breast cancer cases
Source: NPJ Breast Cancer. 2018 Jun 25;4:13. doi: 10.1038/s41523-018-0067-5 (PMC6018637; doi:10.1038/s41523-018-0067-5)
Supplement: Supplementary file 1 — Supplemental Tables 1-3 [file 41523_2018_67_MOESM1_ESM.pdf]

Supplemental Table 1. Patient characteristics for CBCS3 participants with TP53 IHC and NanoString (RNA) compared to all CBCS3 participants

		IHC (N, %[1])	RNA (N, %[1])	All CBCS3 (N, %[1])
Total		1,292	1,013	2,998
Age				
	≤50 years	606 (28.4)	510 (31.0)	1,553 (32.0)
	>50 years	686 (71.6)	503 (69.0)	1,446 (68.0)
	p-value[2]	0.02	0.59	
	p-value[3]	0.18		
Race				
	White	659 (78.1)	517 (77.7)	1,504 (77.5)
	Black	633 (21.9)	496 (22.3)	1,495 (22.5)
	p-value[2]	0.66	0.90	
	p-value[3]	0.81		
Menopausal Status				
	Pre	534 (26.2)	443 (27.9)	1,344 (29.1)
	Post	758 (73.8)	570 (72.1)	1,655 (70.9)
	p-value[2]	0.07	0.50	
	p-value[3]	0.38		
Stage				
	I, II	1,107 (87.9)	841 (84.4)	2,427 (84.7)
	III, IV	169 (12.1)	166 (15.6)	536 (15.3)
	Missing	16	6	36
	p-value[2]	0.02	0.86	
	p-value[3]	0.05		
Node Status				
	Positive	472 (33.0)	426 (39.7)	1,213 (36.0)
	Negative	816 (67.0)	584 (60.3)	1,776 (64.0)
	Missing	4	3	10
	p-value[2]	0.12	0.09	
	p-value[3]	0.01		
Tumor Size				
	≤2cm	729 (63.2)	505 (56.1)	1,513 (58.7)
	>2cm	531 (36.8)	496 (43.9)	1,410 (41.3)
	Missing	32	12	76
	p-value[2]	0.02	0.25	
	p-value[3]	0.01		
Tumor Grade				
	Low-Intermediate	685 (65.1)	494 (59.0)	1,503 (64.8)
	High	527 (34.9)	489 (41.0)	1,150 (35.2)
	Missing	80	30	346
	p-value[2]	0.87	0.01	
	p-value[3]	0.02		
ER Status				
	Negative	343 (22.0)	307 (24.9)	889 (24.4)
	Positive	918 (78.0)	694 (75.1)	2,044 (75.6)
	Missing	31	12	66
	p-value[2]	0.14	0.80	
	p-value[3]	0.17		
PR Status				
	Negative	539 (28.5)	446 (39.2)	1,332 (40.4)
	Positive	717 (61.5)	548 (60.8)	1,594 (59.6)
	Missing	36	19	73
	p-value[2]	0.32	0.61	
	p-value[3]	0.76		
HER2 Status				
	Negative	1,072 (87.4)	848 (88.2)	2,442 (85.8)
	Positive	171 (12.6)	139 (11.8)	441 (14.2)
	Missing	49	26	116
	p-value[2]	0.24	0.11	
	p-value[3]	0.66		
Triple Negative				
	No	967 (82.5)	737 (79.8)	2,184 (81.3)
	Yes	270 (17.4)	245 (20.2)	687 (18.7)
	Missing	55	31	128
	p-value[2]	0.38	0.39	
	p-value[3]	0.15		

[1]Percentages weighted for sampling design
[2]Wald chi-square p-value comparing participants within each TP53 mutation status determination method to all CBCS3 participants
[3]Wald chi-square p-value comparing participants in TP53 IHC to those with NanoString gene expression

Supplemental Table 2. Agreement between TP53 status as determined by IHC and RNA, CBCS3

Overall		
	RNA	
IHC	TP53 wild type-like	TP53 mutant-like
TP53 wild type	400 (70.0)	223 (30.0)
TP53 mutant	41 (22.3)	179 (77.7)
ER+		
	RNA	
IHC	TP53 wild type-like	TP53 mutant-like
TP53 wild type	376 (78.4)	134 (21.6)
TP53 mutant	33 (48.7)	43 (51.3)
ER-		
	RNA	
IHC	TP53 wild type-like	TP53 mutant-like
TP53 wild type	16 (14.2)	87 (85.8)
TP53 mutant	8 (4.4)	134 (95.6)

[1]All percentages weighted for sampling design

Supplemental Table 3. Proportions of mutant TP53 by race and age within strata of hormone receptor positivity, CBCS3

TP53 status	All cases N (%)	Black, ≤50 years N (%)	Black, >50 years N (%)	White, ≤50 years N (%)	White, >50 years N (%)	X^2 p-value
**IHC**						
ER+ or PR+/HER2- & TP53 mutant	90 (11.8)	11 (7.2)	23 (12.6)	28 (13.7)	28 (11.5)	0.20
ER-/PR-/HER2- & TP53 mutant	158 (56.4)	52 (63.7)	57 (60.0)	24 (46.5)	25 (56.8)	<0.01
**RNA-based**						
ER+ or PR+/HER2- & TP53 mutant-like	152 (20.9)	44 (39.9)	42 (32.3)	35 (19.3)	31 (17.5)	<0.01
ER-/PR-/HER2- & TP53 mutant-like	224 (92.0)	75 (95.0)	71 (88.8)	41 (87.2)	37 (94.8)	<0.01

[1]All percentages weighted for sampling design.
